# Phonon frequency comb close to an isolated Einstein mode in $\hbox {InSiTe}_{3}$

**DOI:** 10.1038/s41598-026-44212-1

**Published:** 2026-03-17

**Authors:** Tea Belojica, Jovan Blagojević, Sanja Djurdjić Mijin, Andrijana Šolajić, Jelena Pešić, Emil S. Bozin, Bojana Višić, Yu Liu, Cedomir Petrovic, Zoran V. Popović, Rudi Hackl, Ana Milosavljević, Nenad Lazarević

**Affiliations:** 1 Center for Solid State Physics and New Materials, Institute of Physics Belgrade, University of Belgrade, Pregrevica 118, Belgrade, 11080 Serbia; 2 Departamento de Física de Materiales, Facultad de Ciencias, Universidad Autónoma de Madrid, 28049 Madrid, Spain; 3 Department of Condensed Matter Physics, Jozef Stefan Institute, Jamova cesta 39, 1000 Ljubljana, Slovenia; 4 Shanghai Advanced Research in Physical Sciences (SHARPS), Shanghai, 201203 China; 5 Center for Correlated Matter and School of Physics, Zhejiang University, Hangzhou, 310058 People’s Republic of China; 6 Center for High Pressure Science and Technology Advanced Research (HPSTAR), Beijing, 100094 China; 7 Condensed Matter Physics and Materials Science Department, Brookhaven National Laboratory, Upton, NY 11973-5000 USA; 8 Department of Nuclear and Plasma Physics, Vinca Institute of Nuclear Sciences, University of Belgrade, Belgrade, 11001 Serbia; 9 Serbian Academy of Sciences and Arts, Kneza Mihaila 35, Belgrade, 11000 Serbia; 10 School of Natural Sciences, Department of Physics E51, Technische Universität München, 85748 Garching, Germany; 11 IFW Dresden, Helmholtzstrasse 20, 01069 Dresden, Germany

**Keywords:** Materials science, Physics

## Abstract

The emergence of phonon frequency combs exemplifies a rare and intriguing phenomenon in quantum solids. Materials with distinctive phonon band structures are especially promising for hosting such states, as their vibrational dispersion landscape across the Brillouin zone can facilitate the formation of long-lived, collective lattice excitations. In the layered Van der Waals compound $\textrm{InSi}\textrm{Te}_{3}$, polarization-resolved Raman spectroscopy reveals a pronounced anharmonicity in symmetry-predicted modes and the formation of a self-organized frequency domain structure (coherent-like state), in the range of a localized high-energy $A_{1g}$ phonon mode near 500 cm^−1^. This strong phonon-phonon coupling manifests itself as an anomalous temperature dependence around 200 K, coinciding with the appearance of higher-order excitations within the phonon density of states gap. These findings position $\textrm{InSi}\textrm{Te}_{3}$ as an unconventional platform where intrinsic highly structured phonon spectral correlations and unusually strong anharmonic effects coexist, opening new avenues for exploring emergent vibrational phenomena in low-dimensional materials.

**Supplementary Information:**

The online version contains supplementary material available at 10.1038/s41598-026-44212-1.

## Introduction

Femtosecond pump–probe spectroscopy has long been the primary tool for generating and studying coherent phonons. In these experiments, an ultrashort laser pulse excites the lattice, initiating collective vibrational motion that can be tracked in real time.^1–4^ When a femtosecond laser pulse reshapes the electronic potential, the equilibrium atomic positions are displaced, leaving the atoms to oscillate coherently around the new minimum. This displacive excitation mechanism inherently favors fully symmetric modes. As a result, materials like bismuth and antimony consistently exhibit strong $A_{1g}$ coherent phonons in pump–probe experiments, while lower-symmetry modes appear only under special conditions.^5–7^ In contrast, finding equidistant closely separated phonon lines or a phonon comb without femtosecond excitation is unusual and reflects a nonlinear lattice potential.

Layered Van der Waals (VdW) materials, with their quasi–low-dimensional character, provide an important platform for studying emergent lattice, electronic, and magnetic phenomena.^8,9^ The class is broad, from transition metal trihalides ($\hbox {CrI}_3$, $\hbox {VI}_3$),^10–14^ transition metal trichalcogenides ($\hbox {CrSiTe}_3$, $\hbox {CrGeTe}_3$),^15–18^ iron-based tellurides ($\hbox {Fe}_3$ $\hbox {GeTe}_2$)^19^ to transition metal dichalcogenides ($\hbox {VSe}_2$, $\hbox {MnSe}_2$).^20,21^ The acceleration in the discovery of such quasi-2D systems provides many opportunities to study phonon correlations and nonlinear phenomena as an intrinsic property of the lattice and at the same time to consider their potential for future applications.^22–25^ During the course of this research, Chen et al. reported the spontaneous formation of phonon frequency combs in $\hbox {CrSiTe}_3$ and $\hbox {CrGeTe}_3$,^26^ emphasizing the growing interest in the vibrational properties of ternary VdW trichalcogenides. These developments further emphasize the relevance of exploring related compounds such as $\hbox {InSiTe}_3$, where nonlinearities of the lattice potential may entail unconventional vibrational dynamics.

The first report of $\textrm{InSi}\textrm{Te}_{3}$ single-crystal synthesis appeared more than three decades ago.^27^ Since then, its related compounds such as $\hbox {CrSiTe}_3$, $\hbox {CrGeTe}_3$, $\hbox {InGeTe}_3$, and $\hbox {AlSiTe}_3$ have been extensively studied, both theoretically and experimentally, for their magnetic, electronic, and vibrational properties.^28–33^ By contrast, only a few studies have addressed the fundamental properties of $\textrm{InSi}\textrm{Te}_{3}$.^28,29,34,35^ Nevertheless, it has already been applied in broadband photodetectors with ultrafast response times^36^ which makes a deeper understanding of its lattice dynamics particularly relevant. Low-temperature experiments have not been performed on $\textrm{InSi}\textrm{Te}_{3}$, leaving certain aspects of the physical phenomena below room temperature unexplored.

In this work, we investigate the lattice dynamics of the VdW compound $\hbox {InSiTe}_3$ using temperature-dependent polarization-resolved Raman scattering combined with density functional theory (DFT) calculations. Our study reveals a pronounced anharmonicity and the formation of frequency-domain phonon comb associated with a localized high-energy $A_{1g}$ mode near 500 cm$^{-1}$. This state reflects a frequency-locked spectral response of the $\hbox {SiTe}_3$ tetrahedra, linked to a flat and isolated phonon branch. The temperature-dependent analysis further uncovers anomalous linewidth behavior around 200  K, together with large values of $A_{1g}$ phonon coupling constants. Upon heating to this temperature, broad features emerge in the parallel scattering configuration within the gap of the calculated phonon density of states (PDOS), consistent with overtone excitations. These findings identify $\hbox {InSiTe}_3$ as an important member of the of VdW family and underscore the role of anharmonic interactions in VdW trichalhogenides.

## Results

**Fig. 1:**
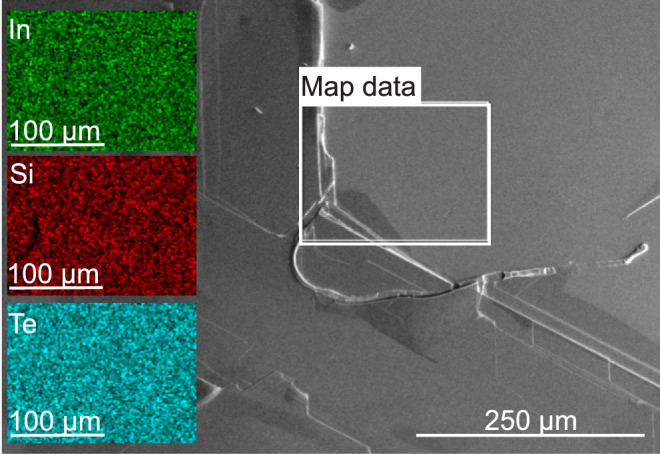
SEM and EDS mapping of a freshly cleaved surface of an $\textrm{InSi}\textrm{Te}_{3}$ single crystal. The right part of the figure shows a flat surface over an extended area. The white rectangle indicates the area in which the EDS mapping was performed. The green, red, and turquoise areas on the left demonstrate the homogeneous distributions of the elements.

The Raman measurements were performed on freshly cleaved surfaces. As shown in Fig. 1 scanning electron microscopy (SEM) reveals a flat surface of at least $0.5\times 0.5~\textrm{mm}^2$. The individual maps obtained with energy dispersive spectroscopy (EDS) as displayed on the left hand side of Fig. 1 demonstrate the uniformity of the sample over several tens of microns and corroborate the atomic ratio of In:Si:Te to be 1:1:3. No impurities, contaminations and vacancies could be detected.

For the phonon analysis, the symmetry positions of the atoms in the lattice are relevant. The factor group analysis for the $P\overline{3}1m$ space group (No. 162)^37^ yields the symmetry allowed phonons in the center of the Brillouin zone (BZ). The corresponding Raman tensors that govern selection rules are listed in Table 1.

In our experiment, the plane of incidence is the $ab$-plane, where $|a| = |b|$ and $\measuredangle (a,b) = 120^\circ$. This plane is oriented perpendicular to the optical axis of the spectrometer, so that the light propagates along the $c$-axis. The incident and scattered light polarizations, $\textbf{e}_\textrm{i}$ and $\textbf{e}_\textrm{s}$, respectively, lie in the $ab$-plane, with $\textbf{e}_\textrm{i} \parallel \textbf{e}_\textrm{s}$ corresponding to $\theta = 0^\circ$ and $\textbf{e}_\textrm{i} \perp \textbf{e}_\textrm{s}$ corresponding to $\theta = 90^\circ$. In these two scattering configurations all allowed Raman active modes ($3A_{1g} + 5E_{g}$) can be observed. The doubly degenerate $E_{g}$ modes are observable in both parallel and crossed polarizations, whereas the fully symmetric $A_{1g}$ modes vanish in configurations with crossed polarization. In addition to the experiments, density-functional theory (DFT) calculations were performed. The resulting frequencies of the optical phonons are in good agreement with the experimental values obtained at 80 K, as summarized in Table 2.

**Table 1:** Atoms, Wyckoff positions, related $\Gamma$ point phonons, and Raman tensors for the $P\overline{3}1m$ space group.

Atoms (Wyckoff positions)	Irreducible representations
In (2*d*)	$A_{2u} + E_{u} + E_{g}$
Si (2*e*)	$A_{2u} + E_{u} + A_{1g} + E_{g}$
Te (6*k*)	$2A_{2u} + 3E_{u} + 2A_{1g} + 3E_{g}$
*Raman tensors*	
$$A_{1g} = \begin{pmatrix} a& 0& 0\\ 0& a& 0\\ 0& 0& b\\ \end{pmatrix}$$	
$$^1E_{g} = \begin{pmatrix} c& 0& 0\\ 0& -c& d\\ 0& d& 0\\ \end{pmatrix}$$	$$^2E_{g} = \begin{pmatrix} 0& -c& -d\\ -c& 0& 0\\ -d& 0& 0\\ \end{pmatrix}$$

**Table 2:** Phonon symmetry, activity, experimental (80 K) and theoretical phonon frequencies (0 K) calculated using experimental crystallographic data. All values are given in cm^−1^. Silent modes are indicated by an asterisk.

Space group: $P\overline{3}1m$ (No. 162)				
Even (*g*)			Odd (*u*)	
Symmetry	Exp.	Calc.	Symmetry	Calc.
$A_{2g} ^{(1)*}$	–	25.5	$A_{2u} ^{(1)}$	0
$E_{g} ^{(1)}$	58.5	57.7	$E_{u} ^1$	0
$E_{g} ^{(2)}$	–	77.0	$A_{2u} ^{(2)}$	67.1
$E_{g} ^{(3)}$	107.8	100.0	$E_{u} ^{(2)}$	69.1
$A_{1g} ^{(1)}$	113.4	108.4	$A_{1u} ^{(1)*}$	89.9
$A_{2g} ^{(2)*}$	–	118.2	$E_{u} ^{(3)}$	92.1
$E_{g} ^{(4)}$	124.9	123.8	$E_{u} ^{(4)}$	111.9
$A_{1g} ^{(2)}$	148.3	145.5	$A_{2u} ^{(3)}$	154.1
$E_{g} ^{(5)}$	–	360.4	$A_{2u} ^{(4)}$	239.4
$A_{1g} ^{(3)}$	498.0	486.4	$E_{u} ^{(5)}$	360.1

**Fig. 2:**
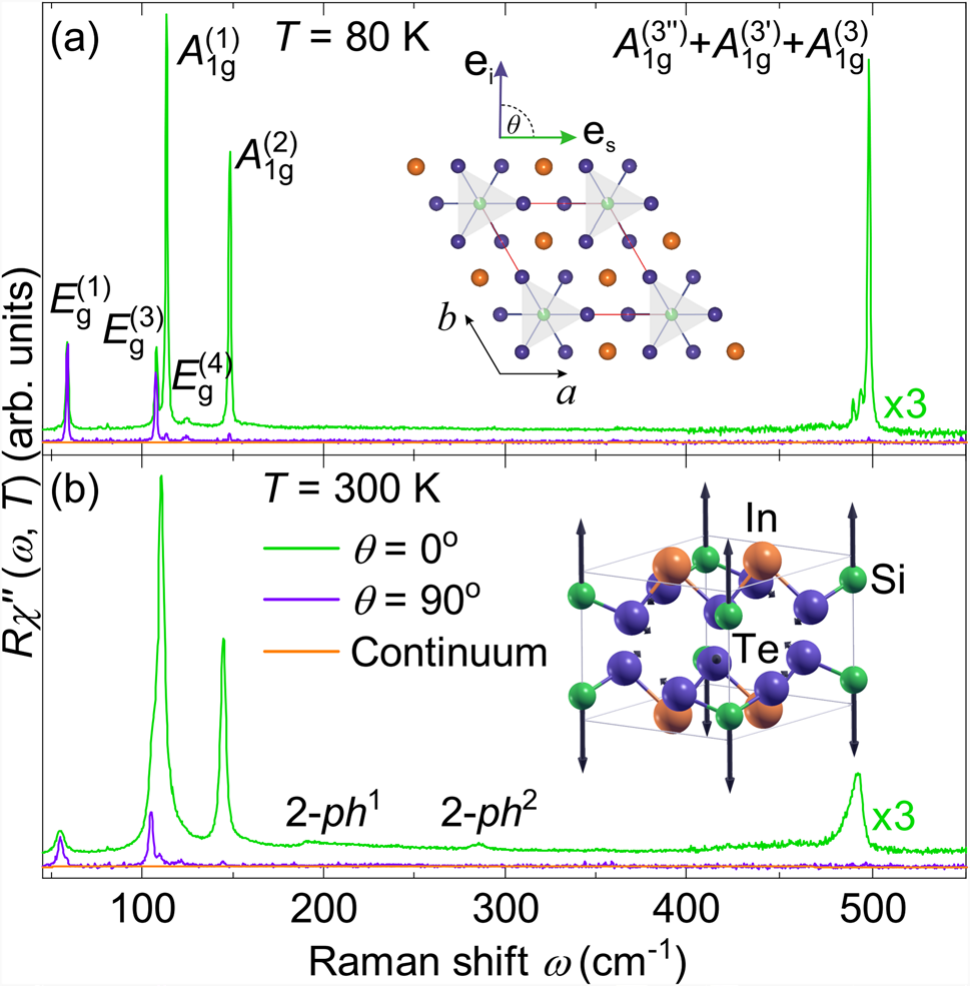
Raman spectra of $\textrm{InSi}\textrm{Te}_{3}$ in parallel ($\theta = 0^{\circ }$) and cross ($\theta = 90^{\circ }$) polarization configurations at (**a**) 80 K and (textbfb) 300 K. The orange lines represent the phenomenological continua (see text). Inset of (**a**) $\textrm{InSi}\textrm{Te}_{3}$ crystallographic unit cell with vectors of incident and scattered light polarizations ${\textbf{e}}_{i}$ and ${\textbf{e}}_{s}$, respectively. For symmetry reasons the orientation of the polarizations with respect to the crystal axes *a* and *b* is irrelevant. Inset of (**b**) displacement pattern of $A_{1g} ^{(3)}$ mode. The arrow lengths are proportional to the square root of the inter-atomic forces. For this mode only the Si atoms move.

Representative Raman spectra of $\textrm{InSi}\textrm{Te}_{3}$ at 80  K and 300  K obtained for both parallel ($\theta = 0$ $^{\circ }$) and crossed ($\theta = 90$ $^{\circ }$) polarization configurations are shown in Fig. 2 (a) and (b), respectively. All three $A_{1g}$ modes and three out of the five expected $E_{g}$ modes appear as narrow lines. At 80 K the Raman spectrum in parallel scattering configuration (Fig. 2a) reveals two extra peaks in the energy range 450 to 500 cm^−1^ labeled as $A^{(3^{\prime \prime })}_{1g}$ and $A^{(3^\prime )}_{1g}$. Although weak in intensity, they are still distinctly observable on the low-energy side of the Raman-active $A_{1g} ^{(3)}$ mode. These additional two peaks and the $A_{1g} ^{(3)}$ phonon are equidistant and persist up to high temperatures, although they cannot be resolved any further as separate lines (Fig. 2b and Fig. 6). As seen in Fig. 2(b), at 300 K additional excitations are observed in the parallel scattering configuration, appearing slightly below 200 cm^−1^ and 300 cm^−1^.

**Fig. 3:**
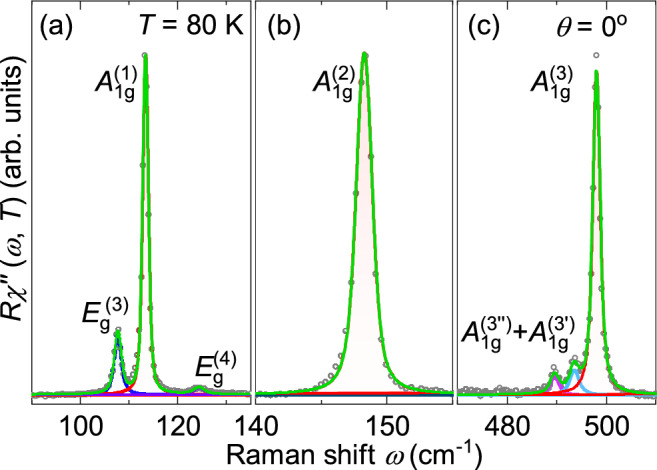
(**a**–**c**) Phonon excitations modeled with Voigt profiles in parallel ($\theta = 0^{\circ }$) polarization configuration where phonons of both $A_{1g}$ and $E_{g}$ symmetry are observed. The spectra are recorded at 80 K.

For the quantitative analysis, the phonon peaks were modeled using a Voigt line shape, defined as the convolution of a Lorentzian and a Gaussian function. The Gaussian component, which accounts for the instrumental resolution of the spectrometer, was fixed to a linewidth of ${\Gamma }_{\textrm{G}}=1$ cm^−1^. To this end, the continuum was modeled phenomenologically using a Drude function together with a linear term: $\chi ^{\prime \prime }_\textrm{continuum}(\omega ) \propto A \Gamma \omega / (\omega ^2 + {\Gamma }^2) + B \omega$ (orange lines in Fig. 2) and was subtracted from the raw data, where *A*, *B*, and $\Gamma$ are temperature dependent phenomenological parameters. The resulting continuum is very weak and essentially constant within the phonon energy range. Fig. 3 displays the results of the phonon analysis in parallel scattering configuration at 80 K.

**Fig. 4:**
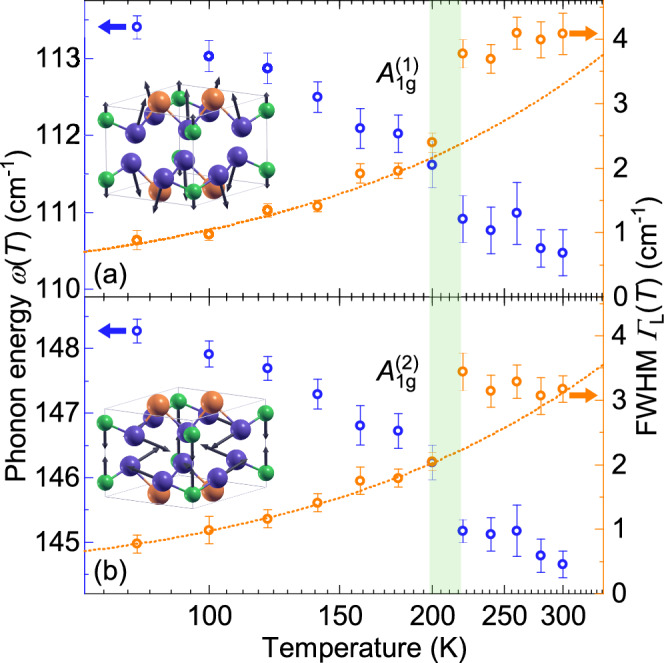
Temperature dependences of the energies and Lorentzian linewidths of the $A_{1g} ^{(1)}$ and $A_{1g} ^{(2)}$ phonons. There are discontinuities of both the energies and linewidths close to 200 K. The dashed lines represent fits to the data below 200 K. The linewidths and energies are well described by anharmonic phonon decay (Eq. 1) and thermal expansion, respectively.

We first focus on the low-lying $A_{1g}$ modes, labeled $A_{1g} ^{(1)}$ and $A_{1g} ^{(2)}$. The temperature dependencies of their energies and linewidths are displayed in Fig. 4, with the vibration patterns shown in insets. For describing the linewidths, we use the Klemens model of symmetric anharmonic decay,^38^

1 $$\begin{aligned} \mathrm {\Gamma _L}(T) = \mathrm {\Gamma _L(0)}\left( 1+\frac{2{\lambda }_{\mathrm {ph-ph}}}{\textrm{e}^{\frac{\hbar {\omega }_{0}}{2\mathrm {k_B}T}}-1}\right) , \end{aligned}$$

where $\mathrm {\Gamma _L(0)}$ and ${\omega }_{0}$ were obtained by extrapolating linewidths and energies to the zero temperature limit. The phonon-phonon coupling parameter ${\lambda _\mathrm {ph-ph}}$ parametrizes the interaction between the optical (here Raman-active) phonon and the acoustical modes at $\pm \textbf{k}$ and $\hbar \omega /2$. The interaction may be mediated by electrons, spins or fluctuations.

At low temperatures $A_{1g} ^{(1)}$ and $A_{1g} ^{(2)}$ exhibit linewidths in the range of $\Gamma _L < 1$ cm^−1^ providing further evidence of the excellent crystal quality. Across the measured temperature range, the linewidths of $A_{1g} ^{(1)}$ and $A_{1g} ^{(2)}$ increase nearly by a factor of four. Up to 200 K the linewidths are well described by the symmetric anharmonic decay as described by Klemens^38^ and described in Eq. 1. The temperature dependence indicates strong phonon–phonon coupling beyond 1, ${\lambda _\mathrm {ph-ph}}\left( A_{1g} ^{(1)}\right) = 1.6$ and ${\lambda _\mathrm {ph-ph}}\left( A_{1g} ^{(2)}\right) = 1.7$.^39^ In both cases, the next data points at 220 K is offset by more than a wavenumber, well beyond the statistical uncertainty. Up to room temperature the linewidths are essentially temperature independent. A similar (and consistent) discontinuity is observed for the energies. The cause of these discontinuities is presently unresolved, with no phase transition reported in this temperature range to the best of our knowledge.

**Fig. 5:**
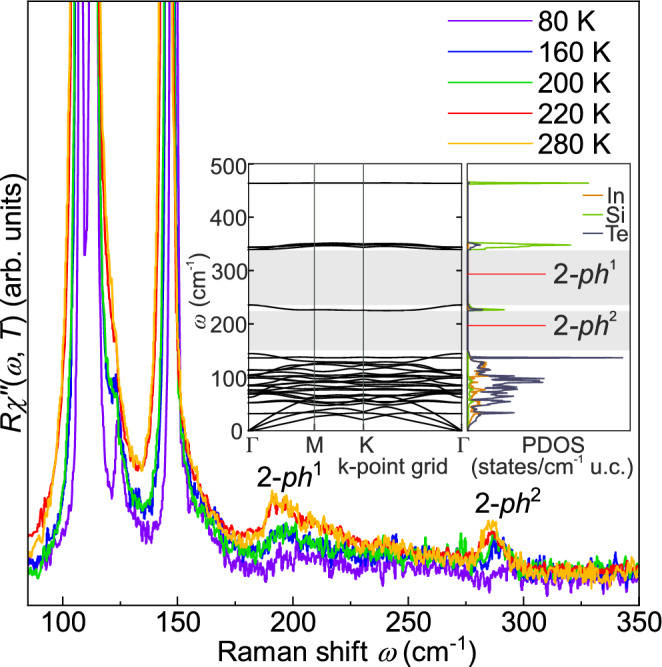
Raman spectra in the range between 80 cm^−1^ and 350 cm^−1^ at temperatures as indicated. The overtone excitations increase abruptly between 200 and 220 K in intensity. Inset: Calculated phonon dispersion along the high-symmetry directions as indicated and PDOS. The shaded area marks the gap in the PDOS.

Even so, the Raman spectra in the range between 80 cm^−1^ and 350 cm^−1^ exhibit an intensity anomaly in the same temperature range as shown in Fig. 5. New features at 200 cm^−1^ and 280 cm^−1^ emerge with increasing temperature. Between 80 and 200 K these broad structures gain intensity slowly and gradually, while they double in intensity between 200 and 220 K and then remain constant up to 300 K. Upon inspecting the phonon dispersion and density of states (PDOS) (see inset of Fig. 5) we conclude that these bands derive from the relatively flat bands in the range of 100 cm^−1^ and the nearly dispersionless band slightly below 150 cm^−1^, mainly deriving from Te vibrations, and may be identified as 2-phonon excitations in the gaps of the phonon bands. Since these 2-phonon bands derive from phonons with energies on the scale of the thermal energy (140 cm^−1^ ≈ 200 K), one may speculate that thermally excited quasiparticles mediate an enhanced coupling between the low-energy Te modes. Then, the $A_{1g} ^{(1)}$ and $A_{1g} ^{(2)}$ Raman modes which just probe this increased coupling get more damped, and the linewidths increase while the energies decrease. Similar as for the Raman-active phonons, the coupling of all other modes increases across the entire Brillouin zone and boosts the two-phonon DOS. This electron-mediated coupling scenario would be consistent with the absence of any signature in the structure.

**Fig. 6:**
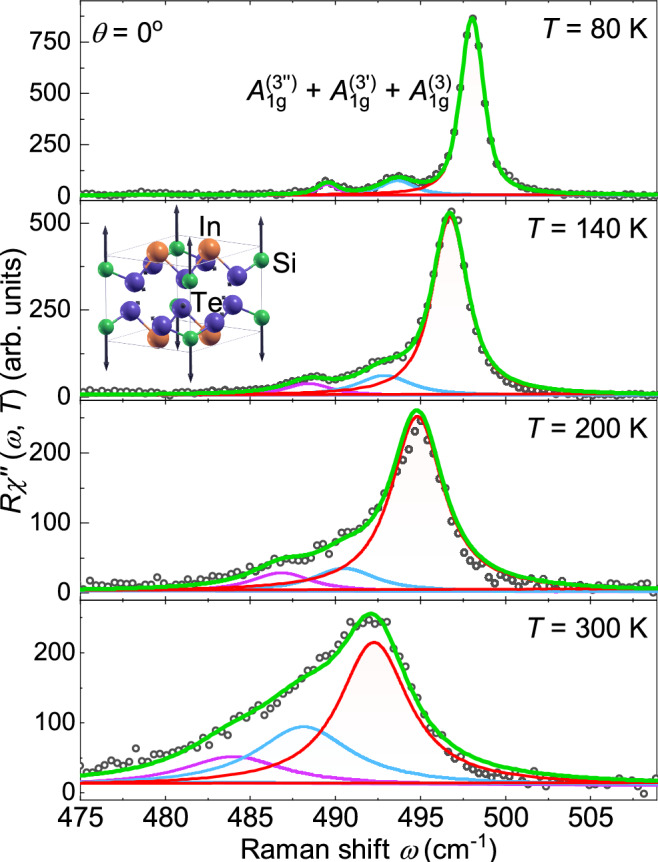
Raman spectra in the range of the $A_{1g} ^{(3)}$ mode at temperatures as indicated. The solid lines represent a Voigt profile fit to the data. All lines become wider with increasing temperature and shift simultaneously to lower energies while maintaining the distance. Inset: Localized vibrations of Te in the $\hbox {SiTe}_3$ tetrahedra associated to the $A_{1g} ^{(3)}$ mode.

**Fig. 7:**
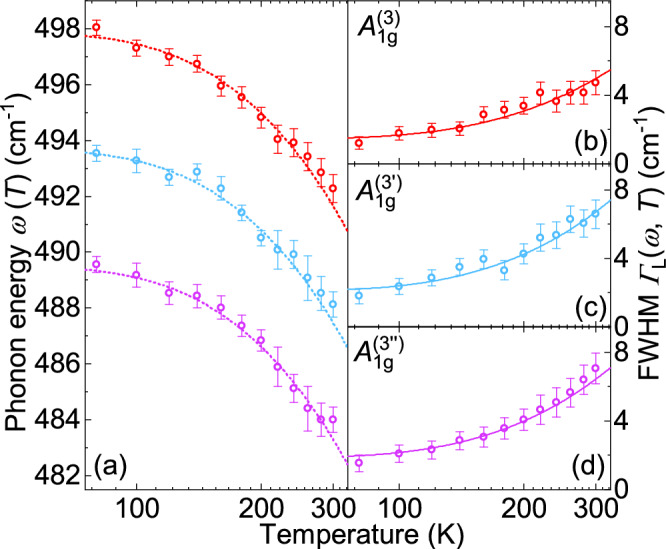
Temperature dependences of energies and linewidths of the $A_{1g} ^{(3)}$ mode and its satellites, $A^{(3^{\prime \prime })}_{1g}$ and $A^{(3^\prime )}_{1g}$ derived from three independent Voigt lineshapes. The equidistant colored dotted lines in (**a**) represent guide to the eye, with the theoretical difference of 4.2 cm^−1^ of neighboring peaks. Solid lines in (**b**) represent the fit of the linewidth data to the anharmonic model (Eq. 1).

The three equidistant lines in the range of the $A_{1g} ^{(3)}$ mode predicted by DFT are fitted with Voigt profiles and shown in Fig. 3. In Fig. 6 they are presented on an expanded energy scale for more temperatures, both along with Voigt profiles and the frequency combs model to be described below. DFT calculations suggest that the $A_{1g} ^{(3)}$ mode is an Einstein phonon with only Si atoms involved far above all other lines (see inset of Fig. 5). However, instead of an isolated line we observe three equidistant lines in the energy range of the $A_{1g} ^{(3)}$ mode. All three $A_{1g} ^{(3)}$ peaks move to lower energy with increasing temperature. While the distances between the modes are essentially temperature independent, the widths increase substantially. Above 200 K the lines cannot be resolved as separate lines in the spectra but only via the fitting procedure (see Fig. 6). When the resulting linewidths and energies are plotted as a function of temperature, no anomalies can be observed, as shown in Fig. 7.

The temperature dependences of all modes are well described by the Klemens model (Fig. 7b–d). The linewidths increase by almost a factor of three over the investigated temperature range. The resulting phonon–phonon coupling parameters, ${\lambda _\mathrm {ph-ph}}\left( A_{1g} ^{(3)}\right) = 2.8$, ${\lambda _\mathrm {ph-ph}}\left( A^{(3^\prime )}_{1g} \right) = 2.5$, and ${\lambda _\mathrm {ph-ph}}\left( A^{(3^{\prime \prime })}_{1g} \right) = 2.8$, are exceptionally large but consistent with the values found recently for phonon combs in $\hbox {CrSiTe}_3$.^26^ The absolute magnitude of ${\lambda _\mathrm {ph-ph}}$ is not universal and may, in addition to the specific decay channels of the Klemens model, depend on the lattice potential, particularly at high temperature, or even on other decay channels.^39–42^ As a matter of fact, the lines become very narrow already at 80 K, the lowest temperature accessible in our experiment, indicating strongly reduced damping. Simultaneously, the lines in the comb become discernible and gain intensity.

**Fig. 8:**
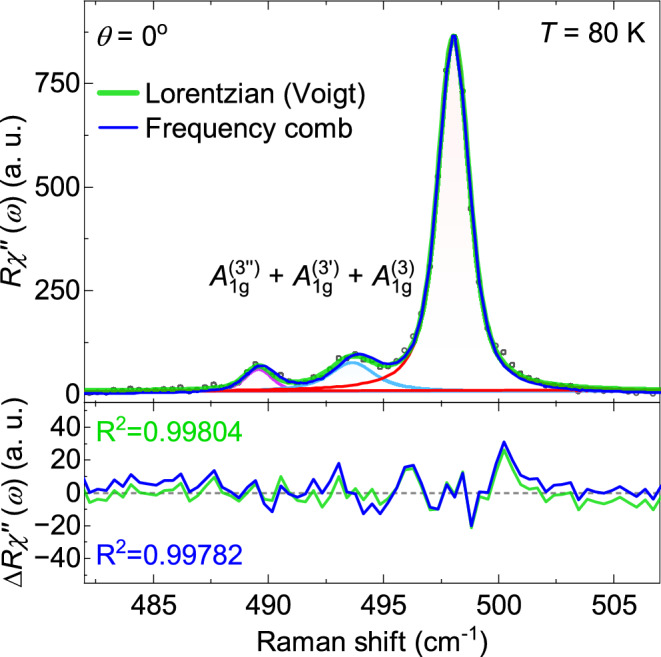
Comparison of coherent-state–based spectral model and individual line model. At 80 K the statistical quality of the frequency comb is only marginally below that of the combination of the three individual lines. (see also Fig. 6 and Supplementary).

On the basis of the increasing intensity and the equidistance of these lines, we employ a coherent-state formalism as a spectral description of the observed frequency-domain features to our spectra as proposed by Chen and coworkers.^26^ Coherent states proposed first by Schrödinger^43^ are widely used as a mathematical framework in superconductivity and laser physics to describe the superposition of a large number of oscillators. In the present context, this approach provides an effective description of the frequency components observed in the Raman spectra but does not imply the existence of a time-dependent coherent phonon expectation value in thermal equilibrium. Rather, it implies the simultaneous excitation of several oscillators^44^ having a very long lifetime. This coherent state is described by an infinite superposition of oscillators,

2 $$\begin{aligned} |\langle x \rangle _\omega |^2=(2\pi )^2 e^{-2|\alpha _0|^2}\sum _{n=0}^{\infty } \frac{|\alpha _0|^{4n+2}}{(n!)^2}[\delta (\omega '-\omega +A+An)+\delta (\omega '-\omega +A-An)], \end{aligned}$$

where $|\alpha _0|$ represents an effective parameter controlling the relative weight of the frequency components in the coherent-state-based spectral description. *A* is proportional to the degree of anharmonicity $\lambda ^2$ in the Hamiltonian (See Supplementary Material). Eq. 2 provides the intensity of the frequency components and describes the distribution of the discrete frequencies $\omega ' = \omega - A - An$ and $\omega ' = \omega - A + An$. To apply this model to describe our data, we replaced the Dirac delta with a Lorentzian having a finite linewidth (convoluted with a Gaussian for the resolution). The extracted energies and linewidths obtained from both the empirical fit and the applied model were nearly identical to within the error margins. A detailed comparison between the two approaches is presented in Fig. 8.

## Discussion

Coherent optical phonons are typically induced by ultrashort pump pulses. They oscillate in phase and produce distinct signatures in optical and Raman spectra.^45^ However, equilibrium Raman spectroscopy probes frequency-domain phonon correlations. If the lattice potential has sufficiently strong anharmonic contributions and the phonons are long-lived, coherent-like spectral responses may arise in the absence of ultrafast excitation.^26^

In the case of $\textrm{InSi}\textrm{Te}_{3}$, the only phonon expected in the energy range around 500 cm^−1^ is a motion of the Si atoms with a negligible involvement of the $\hbox {SiTe}_3$ tetrahedra (Inset of Fig. 6). DFT predicts an isolated high-energy Einstein phonon as shown in the Inset of Fig. 5. However, instead of a single isolated line a series of equidistant peaks is observed in the Raman spectra (Fig. 6), reflecting the underlying nonlinear lattice dynamics. Phonon frequency combs have recently been reported in $\hbox {CrSiTe}_3$ and $\hbox {CrGeTe}_3$.^26^ The comb-like structure was associated with the nonlinear response of a high-energy optical phonon that is spectrally well separated from other phonon states. In $\textrm{InSi}\textrm{Te}_{3}$, the $A_{1g} ^{(3)}$ mode is separated by a sufficiently large gap in PDOS. Because of this spectral isolation, the mode remains long-lived, as the available decay channels are strongly limited. As a consequence equidistant spectral components emerge.

An alternative explanation for persistent equidistant peak structure may be thermal ”hot-band” progression arising from anharmonic vibrational ladders. For an anharmonic potential in second order perturbation theory we get relatively closely spaced energies at distances $\Delta \omega$. The intensity of the *n*-th satellite is expected to scale as $I_n/I_0 \propto \exp (-(\hbar \omega - n\hbar \Delta \omega )/k_B T)$, where $\omega = 498.0$ cm^−1^ corresponds to the energy of the $A_{1g} ^{(3)}$ phonon ($n=0$) at 80  K, and $\Delta \omega = 4.2$ cm^−1^. As shown in Supplementary Fig. S1, the experimentally observed temperature dependences of the sattellites at 493.8 cm^−1^ ($n=1$) and 489.6 cm^−1^ ($n=2$) deviate strongly from this expectation. This discrepancy allows us to exclude a thermal hot-band origin of the observed phonon frequency comb.

In addition, mechanisms based on the thickness or layer dependent quantization can be excluded in the present case. All Raman measurements reported here were performed on bulk single crystals, where the macroscopic sample dimensions prevent the formation of discrete low-energy standing acoustic modes associated with finite-size confinement. In such confined geometries, the characteristic mode spacing is expected to scale with sample thickness or number of layers. No such dependence is observed here.

The satellite peaks observed in $\textrm{InSi}\textrm{Te}_{3}$ are not related to phonon beating either. Phonon beating requires the coexistence of multiple distinct phonon modes with closely spaced frequencies, as demonstrated, for example, for the $A_{1g}$ modes in $\hbox {Cd}_3$ $\hbox {As}_2$ induced by helix vacancies. ^46^ In contrast, for $\textrm{InSi}\textrm{Te}_{3}$ one expects a single isolated $A_{1g} ^{(3)}$ optical phonon branch (Fig. 6) with no nearby modes that could give rise to beating. The observed satellites therefore originate from the nonlinear lattice potential leading to additional eigenstates in second order perturbation theory^26^ rather than independent phonon excitations.

In $\hbox {Cr}_2$ $\hbox {Ge}_2$ $\hbox {Te}_6$ comb-like structures in the phonon spectra were observed recently and interpreted in terms isotope shifts. ^47^ In $\textrm{InSi}\textrm{Te}_{3}$, the isotope satellites were to be expected at 9 cm^−1^ and 17 cm^−1^ below the main line at 500 cm^−1^ as opposed to 4.2 and 8.4 cm^−1^ here, and their intensities and linewidths exhibit a systematic temperature evolution different from that expected for isotopes.

Finally, we wish to address the dilemma of the strong phonon-phonon coupling of the 500 cm^−1^ modes derived via the Klemens model (Eq. 1), suggesting a rapid decay, and the long lifetime required for phase coherence. As already pointed out above symmetric decay is not the only mechanism for line broadening. In an anharmonic potential the lines may get broader also by a stronger shift of a the resonance frequencies at elevated temperatures and a superposition of lines at different energies. At low temperatures there are barely any channels for the isolated Einstein mode (and possible satellites) to decay into low-energy phonons as obvious from the DFT phonon dispersion (Inset of Fig. 5). The separation from the continuum of other vibrational states by a sufficiently large gap in the PDOS quenches all decay channels for symmetric decay. We conclude that the distortion of the harmonic potential required for the formation of the observed frequency comb does not originate from the anharmonic decay of the Einstein-like mode.

The temperature dependences of the fully symmetric low-energy peaks, $A_{1g}^{(1)}$ and $A_{1g}^{(2)}$, change discontinuously in the vicinity of 200 K (Fig. 4). Specifically the line widths do not follow the expected anharmonic variation any longer, indicating a change in phonon–phonon coupling. Along with these discontinuities at 200 K, two-phonon excitations (see Fig. 5) become more pronounced. In the Raman spectra, structures beyond the allowed lines may originate either from defect-induced scattering, in which case the spectral features follow a projected PDOS, or from interactions in the material.^39^ A comparison of the spectra with the calculated PDOS, in addition to the SEM analysis allow us to rule out the defect scenario. The fact that the two-phonon excitations appear exclusively in the $A_{1g}$ channel is consistent with the selection rules for two-phonon scattering, where the direct product of two phonon symmetries always contains the totally symmetric representation.^39^ The unusually strong coupling of the $A_{1g}$ modes in $\textrm{InSi}\textrm{Te}_{3}$ further enhances multi-phonon scattering in this channel, making the overtone features particularly pronounced. As the thermal phonon population and electron occupation increase, these higher-order processes become more probable and the overtones emerge clearly within the PDOS gap. By comparing their positions with the calculated phonon dispersion, the two-phonon features can be linked to low-energy PDOS maxima at roughly half the energies of 2-$ph^1$ and 2-$ph^2$ ($\approx 95$ and $\approx 145$ cm^−1^, respectively), indicating contributions from acoustic or low-lying optical branches (Inset of Fig. 5).

These observations reveal that the lattice dynamics of $\textrm{InSi}\textrm{Te}_{3}$ are dominated by strong phonon–phonon interactions and give rise to pronounced anomalies near 200 K. Although a microscopic model for these features is still lacking, we propose that the thermal population of phononic and electronic states in this narrow-gap semiconductor drives these interactions. The appearance of overtone excitations within a PDOS gap supports this interpretation. The formation of a frequency comb, generated by coherent-like phonons and persisting across the full temperature range studied, suggests that the well-isolated Einstein phonon in $\textrm{InSi}\textrm{Te}_{3}$ plays a central role in stabilizing highly structured vibrational spectrum. Together with recent reports of coherent-like phonon states in $\hbox {CrSiTe}_3$ and $\hbox {CrGeTe}_3$,^26^ our findings position Van der Waals trichalcogenides as promising platforms for discovering frequency combs in phonon spectra.

## Methods

### Experimental methods

Single crystals of $\textrm{InSi}\textrm{Te}_{3}$ were synthesized by melting stoichiometric mixture of In (5N, Alfa Aesar) chunk, Si (5N, Alfa Aesar) lump, and Te (5N, Alfa Aesar) shot. The starting materials were vacuum-sealed in a quartz tube, heated to 1100 $^{\circ }$C over 20 hours, held at 1100 $^{\circ }$C for 12 hours and then cooled to 700 $^{\circ }$C at rate of 1 $^{\circ }$C/h.

Scanning electron microscopy was performed using FEI HeliosNanolab 650 scanning electron microscope (SEM) equipped with an Oxford Instruments energy dispersive spectroscopy (EDS) system with an X-max SSD detector operating at 20 kV. Measurements were performed on as-cleaved samples deposited on a graphite tape. To determine the presence of different elements, an area about the size of 100 $\times$ 100 $\mu$m was selected on the deposited material where the EDS analysis was performed.

Inelastic scattering measurements of $\textrm{InSi}\textrm{Te}_{3}$ were conducted using Tri Vista 557 Raman spectrometer in the subtractive backscattering configuration, with the combination of gratings 1800/1800/2400 grooves/mm. As an excitation source Ar$^+$/Kr$^+$ ion laser with 514  nm line was used. In this scattering configuration plane of incidence is *ab* plane ($\vert {a}\vert = \vert {b}\vert$, $\measuredangle (a,b) =$120$^{\circ }$) with incident/scattered light propagation direction along crystallographic *c*-axis. The samples were cleaved right before being placed in a high vacuum ($10^{-6}$ mbar), which was achieved with a KONTI CryoVac continuous Helium Flow cryostat with 0.5 mm thick window. Laser beam focusing was accomplished using microscope objective with $\times$50 magnification to a spot size of approximately $8\,\mu \textrm{m}$. All Raman spectra were corrected for the Bose factor.

### Theoretical methods

First-principles calculations based on density functional theory (DFT) were carried out using the Quantum ESPRESSO package.^48^ The Perdew–Burke–Ernzerhof revised for solids (PBEsol) exchange-correlation functional^49^ within the generalized gradient approximation (GGA) was employed to better describe lattice parameters in crystalline solids. The cutoff for wavefunctions and the charge density were set to 50 Ry and 400 Ry, respectively. The *k*-points were sampled using the Monkhorst-Pack scheme, on a 12$\times$12$\times$12 $\Gamma$ - centered grid. For accurate treatment of interlayer interactions, the Van der Waals interactions is included using the Grimme-D2 correction. Phonon frequencies were calculated with the linear response method, as implemented in Phonon package of the Quantum Espresso. The initial atomic structure of $\textrm{InSi}\textrm{Te}_{3}$ was obtained from an experimental CIF file and used as the starting point for first-principles calculations. According to experimental crystallographic data, $\textrm{InSi}\textrm{Te}_{3}$ exhibits trigonal symmetry and belongs to the $P\overline{3}1m$ (No. 162) space group. All calculations correspond to the harmonic approximation at zero temperature and do not include anharmonic or finite-temperature effects.

## Supplementary Information

Supplementary Information.

## Data Availability

All data supporting the findings of this study are available within the paper and its Supplementary Information. Additional data are available upon reasonable request from the corresponding author.

## References

[1] Zeiger, H. J. et al. Theory for displacive excitation of coherent phonons. *Phys. Rev. B* **45**, 768–778. 10.1103/PhysRevB.45.768 (1992). 10.1103/physrevb.45.768 10001117

[2] Vialla, F. & Fatti, N. D. Time-domain investigations of coherent phonons in van der Waals thin films. *Nanomaterials*10.3390/nano10122543 (2020). 33348750 10.3390/nano10122543 PMC7766349

[3] Misochko, O. V. Coherent phonons and their properties. *J. Exp. Theor. Phys.* **92**, 246–259. 10.1134/1.1354682 (2001).

[4] Melnikov, A. A., Selivanov, Y. G. & Chekalin, S. V. Anharmonic coherent dynamics of the soft phonon mode of a PbTe crystal. *Phys. Rev. B* **108**, 224309. 10.1103/PhysRevB.108.224309 (2023).

[5] Ishioka, K., Kitajima, M. & Misochko, O. V. Coherent A and E phonons of antimony. *J. Appl. Phys.* **103**, 123505. 10.1063/1.2940130 (2008).

[6] Ishioka, K., Kitajima, M. & Misochko, O. V. Temperature dependence of coherent A and E phonons of bismuth. *J. Appl. Phys.* **100**, 093501. 10.1063/1.2363746 (2006).

[7] Garrett, G. A., Albrecht, T. F., Whitaker, J. F. & Merlin, R. Coherent THz phonons driven by light pulses and the Sb problem: What is the mechanism?. *Phys. Rev. Lett.* **77**, 3661–3664. 10.1103/PhysRevLett.77.3661 (1996). 10062276 10.1103/PhysRevLett.77.3661

[8] Lazarević, N. & Hackl, R. Fluctuations and pairing in Fe-based superconductors: Light scattering experiments. *J. Phys.: Condens. Matter* **32**, 413001. 10.1088/1361-648X/ab8849 (2020). 10.1088/1361-648X/ab8849 32272462

[9] Wang, Q. H. et al. The magnetic genome of two-dimensional van der Waals materials. *ACS Nano* **16**, 6960–7079. 10.1021/acsnano.1c09150 (2022) (**PMID: 35442017**). 35442017 10.1021/acsnano.1c09150 PMC9134533

[10] Liu, Y. & Petrovic, C. Three-dimensional magnetic critical behavior in CrI. *Phys. Rev. B* **97**, 014420. 10.1103/PhysRevB.97.014420 (2018).

[11] Huang, B. et al. Layer-dependent ferromagnetism in a van der Waals crystal down to the monolayer limit. *Nature* **546**, 270–273. 10.1038/nature22391 (2017). 28593970 10.1038/nature22391

[12] Wang, Y.-P. & Long, M.-Q. Electronic and magnetic properties of van der Waals ferromagnetic semiconductor VI. *Phys. Rev. B* **101**, 024411. 10.1103/PhysRevB.101.024411 (2020).

[13] Djurdjić Mijin, S. et al. Short-range order in VI. *Inorg. Chem.* **59**, 16265–16271, 10.1021/acs.inorgchem.0c02060 (2020). 10.1021/acs.inorgchem.0c02060 33092339

[14] Djurdjić Mijin, S. et al. Lattice dynamics and phase transition in single crystals. *Phys. Rev. B* **98**, 104307. 10.1103/PhysRevB.98.104307 (2018).

[15] Ouvrard, G., Sandre, E. & Brec, R. Synthesis and crystal structure of a new layered phase: The chromium hexatellurosilicate CrSiTe. *J. Solid State Chem.* **73**, 27–32. 10.1016/0022-4596(88)90049-7 (1988).

[16] Zhang, X. et al. Magnetic anisotropy of the single-crystalline ferromagnetic insulator CrGeTe. *Jpn. J. Appl. Phys.* **55**, 033001. 10.7567/jjap.55.033001 (2016).

[17] Milosavljević, A. et al. Evidence of spin-phonon coupling in . *Phys. Rev. B* **98**, 104306. 10.1103/PhysRevB.98.104306 (2018).

[18] Milosavljević, A. et al. Vacancies and spin–phonon coupling in CrSiGeTe. *J. Raman Spectrosc.* **51**, 2153–2160, 10.1002/jrs.5962 (2020).

[19] Fei, Z. et al. Two-dimensional itinerant ferromagnetism in atomically thin FeGeTe. *Nat. Mater.* **17**, 778–782. 10.1038/s41563-018-0149-7 (2018). 30104669 10.1038/s41563-018-0149-7

[20] Bonilla, M. et al. Strong room-temperature ferromagnetism in VSe monolayers on van der Waals substrates. *Nat. Nanotechnol.* **13**, 289–293. 10.1038/s41565-018-0063-9 (2018). 29459653 10.1038/s41565-018-0063-9

[21] O’Hara, D. J. et al. Room temperature intrinsic ferromagnetism in epitaxial manganese selenide films in the monolayer limit. *Nano Lett.* **18**, 3125–3131. 10.1021/acs.nanolett.8b00683 (2018). 29608316 10.1021/acs.nanolett.8b00683

[22] Sierra, J. F., Fabian, J., Kawakami, R. K., Roche, S. & Van der Valenzuela, S. O. Waals heterostructures for spintronics and opto-spintronics. *Nat. Nanotechnol.*10.1038/s41565-021-00936-x (2021). 34282312 10.1038/s41565-021-00936-x

[23] Yang, S., Zhang, T. & van der Jiang, C. Waals magnets: Material family, detection and modulation of magnetism, and perspective in spintronics. *Adv. Sci.* **8**, 2002488. 10.1002/advs.202002488 (2021). 10.1002/advs.202002488 PMC7816723 33511010

[24] Glavin, N. R. et al. Emerging applications of elemental 2D materials. *Adv. Mater.* **32**, 1904302. 10.1002/adma.201904302 (2020). 10.1002/adma.201904302 31667920

[25] Khan, K. et al. Recent advances in two-dimensional materials and their nanocomposites in sustainable energy conversion applications. *Nanoscale* **11**, 21622–21678. 10.1039/C9NR05919A (2019). 31702753 10.1039/c9nr05919a

[26] Chen, L. et al. Spontaneously formed phonon frequency combs in van der Waals solid CrGeTe and CrSiTe. *Nat. Commun.* **16**, 5795. 10.1038/s41467-025-61173-7 (2025). 40592915 10.1038/s41467-025-61173-7 PMC12216165

[27] Sandre, E., Carteaux, V., Marie, A.M. & Ouvrard, G. ChemInform abstract: A new lamellar Tellurosilicate, InSiTe. *ChemInform*, **23**, 10.1002/chin.199234029 (1992).

[28] Korkmaz, M. A., Deligoz, E. & Ozisik, H. Strong elastic anisotropy of low-dimensional ternary compounds: InXTe (X = Si, Ge). *J. Electron. Mater.* **50**, 2779–2788. 10.1007/s11664-021-08784-0 (2021).

[29] Lefevre, R. et al. Layered tellurides: Stacking faults induce low thermal conductivity in the new InGeTe and thermoelectric properties of related compounds. *J. Mater. Chem. A* **5**, 19406–19415. 10.1039/C7TA04810F (2017).

[30] Sandre, E., Carteaux, V., Marie, A. M. & Ouvrard, G. Structural determination of a new lamellar tellurosilicate, AlSiTe. *J. Alloy. Compd.* **204**, 145–149. 10.1016/0925-8388(94)90083-3 (1994).

[31] Casto, L. D. et al. Strong spin-lattice coupling in CrSiTe. *APL Mater.* **3**, 041515. 10.1063/1.4914134 (2015).

[32] Bhoi, D. et al. Nearly room-temperature ferromagnetism in a pressure-induced correlated metallic state of the van der Waals insulator . *Phys. Rev. Lett.* **127**, 217203. 10.1103/PhysRevLett.127.217203 (2021). 34860097 10.1103/PhysRevLett.127.217203

[33] Watson, M. D. et al. Direct observation of the energy gain underpinning ferromagnetic superexchange in the electronic structure of . *Phys. Rev. B* **101**, 205125. 10.1103/PhysRevB.101.205125 (2020).

[34] Suriwong, T., Kurosaki, K. & Thongtem, S. Thermoelectric properties of phosphorus-doped indium tellurosilicate: InSiTe. *J. Alloy. Compd.* **735**, 75–80. 10.1016/j.jallcom.2017.11.093 (2018).

[35] Debbichi, L., Kim, H., Björkman, T., Eriksson, O. & Lebègue, S. First-principles investigation of two-dimensional trichalcogenide and sesquichalcogenide monolayers. *Phys. Rev. B* **93**, 245307. 10.1103/PhysRevB.93.245307 (2016).

[36] Chen, J. et al. A submicrosecond-response ultraviolet–visible–near-infrared broadband photodetector based on 2D tellurosilicate InSiTe. *ACS Nano* **16**, 7745–7754. 10.1021/acsnano.1c11628 (2022) (**PMID: 35499232**). 35499232 10.1021/acsnano.1c11628

[37] Jin, Y., Wang, R. & Xu, H. Recipe for Dirac phonon states with a quantized valley berry phase in two-dimensional hexagonal lattices. *Nano Lett.* **18**, 7755–7760. 10.1021/acs.nanolett.8b03492 (2018). 30456958 10.1021/acs.nanolett.8b03492

[38] Klemens, P. G. Anharmonic decay of optical phonons. *Phys. Rev.* **148**, 845–848. 10.1103/PhysRev.148.845 (1966).

[39] Baum, A. et al. Phonon anomalies in FeS. *Phys. Rev. B* **97**, 054306. 10.1103/PhysRevB.97.054306 (2018).

[40] Kumar, D., Kumar, V., Kumar, R., Kumar, M. & Kumar, P. Electron-phonon coupling, thermal expansion coefficient, resonance effect, and phonon dynamics in high-quality CVD-grown monolayer and bilayer . *Phys. Rev. B* **105**, 085419. 10.1103/PhysRevB.105.085419 (2022).

[41] Tiwari, A. et al. Spin-phonon-charge coupling in the two-dimensional honeycomb lattice compound . *Phys. Rev. B* **108**, 075113. 10.1103/PhysRevB.108.075113 (2023).

[42] Poojitha, B., Shaji, A., Badola, S. & Saha, S. Spin–phonon coupling in ferrimagnet spinel CoMnO. *J. Chem. Phys.* **156**, 184701. 10.1063/5.0087770 (2022). 35568549 10.1063/5.0087770

[43] Schrödinger, E. Der. stetige Übergang von der Mikro-zur Makromechanik. *Naturwissenschaften* **14**, 664–666 (1926).

[44] Martin, R. M. & Varma, C. M. Cascade theory of inelastic scattering of light. *Phys. Rev. Lett.* **26**, 1241–1244. 10.1103/PhysRevLett.26.1241 (1971).

[45] Zhai, Y., Gong, P., Hasaien, J., Zhou, F. & Zhao, J. Coherent phonons in correlated quantum materials. *Prog. Surf. Sci.* **99**, 100761. 10.1016/j.progsurf.2024.100761 (2024).

[46] Sun, F. et al. Coherent helix vacancy phonon and its ultrafast dynamics waning in topological Dirac semimetal . *Phys. Rev. B* **95**, 235108. 10.1103/PhysRevB.95.235108 (2017).

[47] Krasucki, G. et al. Spin-phonon coupling and isotope-related pseudo-molecule vibrations in layered CrGeTe ferromagnet (2025). arXiv: 2510.01881.

[48] Giannozzi, P. et al. Quantum espresso: A modular and open-source software project for quantum simulations of materials. *J. Phy. Condens. Mat.* **21**, 395502. 10.1088/0953-8984/21/39/395502 (2009). 10.1088/0953-8984/21/39/395502 21832390

[49] Perdew, J. P., Burke, K. & Ernzerhof, M. Generalized gradient approximation made simple. *Phys. Rev. Lett.* **77**, 3865–3868. 10.1103/PhysRevLett.77.3865 (1996). 10062328 10.1103/PhysRevLett.77.3865

