# Supplementary material for: Phonon frequency comb close to an isolated Einstein mode in [image]
Source: Sci Rep. 2026 Mar 17;16:13944. doi: 10.1038/s41598-026-44212-1 (PMC13133099; doi:10.1038/s41598-026-44212-1)
Supplement: Supplementary file 1 — Supplementary Information. [file 41598_2026_44212_MOESM1_ESM.pdf]

Supplementary information
Phonon frequency comb close to an isolated Einstein mode in $InSiTe_3$

Tea Belojica[1], Jovan Blagojević[1], Sanja Djurdjić Mijin[1,2], Andrijana Šolajić[1], Jelena Pešić[1], Emil S. Bozin[1], Bojana Višić[1,3], Yu Liu[4,5], Cedomir Petrovic[4,6,7,8], Zoran V. Popović[9], Rudi Hackl[10,11], Ana Milosavljević[1,*], and Nenad Lazarević[1]

[1]Center for Solid State Physics and New Materials, Institute of Physics Belgrade, University of Belgrade, Pregrevica 118, 11080 Belgrade, Serbia
[2]Departamento de Física de Materiales, Facultad de Ciencias, Universidad Autónoma de Madrid, 28049 Madrid, Spain
[3]Department of Condensed Matter Physics, Jozef Stefan Institute, Jamova cesta 39, Ljubljana 1000, Slovenia
[4]Shanghai Advanced Research in Physical Sciences (SHARPS), Shanghai 201203, China
[5]Center for Correlated Matter and School of Physics, Zhejiang University, Hangzhou 310058, People's Republic of China
[6]Center for High Pressure Science & Technology Advanced Research (HPSTAR) - Beijing 100094, China
[7]Condensed Matter Physics and Materials Science Department, Brookhaven National Laboratory, Upton, NY 11973-5000, USA
[8]Department of Nuclear and Plasma Physics, Vinca Institute of Nuclear Sciences, University of Belgrade, Belgrade 11001, Serbia
[9]Serbian Academy of Sciences and Arts, Kneza Mihaila 35, 11000 Belgrade, Serbia
[10]School of Natural Sciences, Department of Physics E51, Technische Universität München, 85748 Garching, Germany
[11]IFW Dresden, Helmholtzstrasse 20, 01069 Dresden, Germany
[*]Corresponding author: ana.milosavljevic@ipb.ac.rs

Calculation of the Raman Intensity

We closely follow the derivation of the coherent-state dynamics presented in Ref. 1. Starting from the anharmonic oscillator Hamiltonian with cubic perturbation $H = \frac{p^2}{2\mu} + \frac{1}{2}\mu\omega^2x^2 + \lambda x^3$, the time evolution of the annihilation operator is obtained as

$$\langle a \rangle_t = \alpha_0 e^{-|\alpha_0|^2} e^{-i(\omega-A)t} \exp(|\alpha_0|^2 e^{iAt}) = \alpha_0 \exp[-i(\omega-A)t + |\alpha_0|^2(e^{iAt}-1)], \tag{1}$$

which directly leads to the displacement expectation value

$$\langle x \rangle_t = \langle a^\dagger + a \rangle_t = e^{-|\alpha_0|^2} \sum_{n=0}^{\infty} \frac{|\alpha_0|^{2n}\alpha_0}{n!} e^{-i(E_{n+1}-E_n)t/\hbar} + \text{c.c.} \tag{2}$$

where c.c. is the complex conjugate [see Ref. 1 and Supplementary Equations (7)–(8)].
To obtain the spectral distribution relevant for the Raman process, we calculate the Fourier transform

$$\langle x \rangle_\omega = \int_{-\infty}^{\infty} \langle x \rangle_t e^{i\omega' t}\, dt. \tag{3}$$

Separating $\langle x \rangle_t$ into $f(t)$ and its complex conjugate, with

$$f(t) = \alpha_0 e^{-i(\omega-A)t+|\alpha_0|^2(e^{iAt}-1)} \tag{4}$$

and expanding the exponential using the Poisson series

$$e^{|\alpha_0|^2 e^{iAt}} = \sum_{n=0}^{\infty} \frac{|\alpha_0|^{2n}}{n!} e^{iAnt}. \tag{5}$$

we obtain

$$f(t) = \alpha_0 e^{-i(\omega - A)t} e^{-|\alpha_0|^2} \sum_{n=0}^{\infty} \frac{|\alpha_0|^{2n}}{n!} e^{iAnt}. \tag{6}$$

Its Fourier transform reads

$$\tilde{f}_\omega = 2\pi \alpha_0 e^{-|\alpha_0|^2} \sum_{n=0}^{\infty} \frac{|\alpha_0|^{2n}}{n!} \delta(\omega' - \omega + A + An). \tag{7}$$

Adding the conjugate contribution, we obtain

$$\langle x \rangle_\omega = 2\pi e^{-|\alpha_0|^2} \sum_{n=0}^{\infty} \frac{|\alpha_0|^{2n}}{n!} [\alpha_0 \delta(\omega' - \omega + A + An) + \alpha_0^* \delta(\omega' - \omega + A - An)]. \tag{8}$$

Finally, the Raman intensity is proportional to

$$I(\omega') \propto |\langle x \rangle_\omega|^2 = (2\pi)^2 e^{-2|\alpha_0|^2} \sum_{n=0}^{\infty} \frac{|\alpha_0|^{4n+2}}{(n!)^2} [\delta(\omega' - \omega + A + An) + \delta(\omega' - \omega + A - An)], \tag{9}$$

which is the expression used in the main text to describe the frequency comb distribution. Here $|\alpha_0|$ represents the amplitude of the coherent state and $A \propto \lambda^2$, with λ from the Hamiltonian's anharmonic term.

In our case, the fitting procedure yields $A = 4.2$ and $\alpha_0 = 2.15$. The relatively large value of A reflects the strong anharmonicity of the $A_{1g}^{(3)}$ vibration, and represents the regular spacing of the comb peaks. Meanwhile, α_0 governs the relative contribution of higher-order frequency components in the spectral model. The obtained value implies that multiple peaks acquire appreciable spectral weight, consistent with the experimentally observed comb structure.

Exclusion of a thermal hot-band mechanism

To inspct whether the equidistant satellite peaks of the $A_{1g}^{(3)}$ mode could originate from a thermally populated vibrational ladder (hot-band progression), we consider an anharmonic potential within second-order perturbation theory. In such a case, the vibrational energy levels are expected to be separated by approximately $\Delta\omega$, where $\Delta\omega$ corresponds to the observed satellite spacing. In the present case, $\Delta\omega \approx 4.2$ cm^{-1}.

We therefore compare the first ($n = 1$) and second ($n = 2$) satellite peaks at 493.8 cm^{-1} and 489.6 cm^{-1} to the main peak ($n = 0$) at $\omega = 498.0$ cm^{-1}, corresponding to the $A_{1g}^{(3)}$ phonon energy at 80 K. Within a thermal hot-band picture, the relative intensity of the n-th satellite is expected to scale as $I_n/I_0 \propto \exp(-(\hbar\omega - n\hbar\Delta\omega)/k_B T)$. Since $\omega \approx 498.0$ cm^{-1} corresponds to an energy scale of approximately 700 K, the hot-band model predicts an exponential suppression of satellite intensities at low temperatures, with a pronounced increase only at elevated temperatures. The relative integrated intensities of the $n = 1$ and $n = 2$ satellites as a function of temperature are shown in Fig. S1 and compared them directly to the corresponding Boltzmann factors. Contrary, well-defined satellite peaks are already observed at temperatures as low as 80 K, where the Boltzmann population of the $A_{1g}^{(3)}$ phonon is negligibly small. Furthermore, the measured temperature dependence of the satellite intensities deviates strongly from the exponential scaling expected for a thermally populated vibrational ladder. This clear disagreement between experiment and the hot-band prediction allows us to exclude this effect as the origin of the observed equidistant peaks.

References

1. Chen, L. *et al.* Spontaneously formed phonon frequency combs in van der Waals solid $CrGeTe_3$ and $CrSiTe_3$. *Nat. Commun.* **16**, 5795, 10.1038/s41467-025-61173-7 (2025).

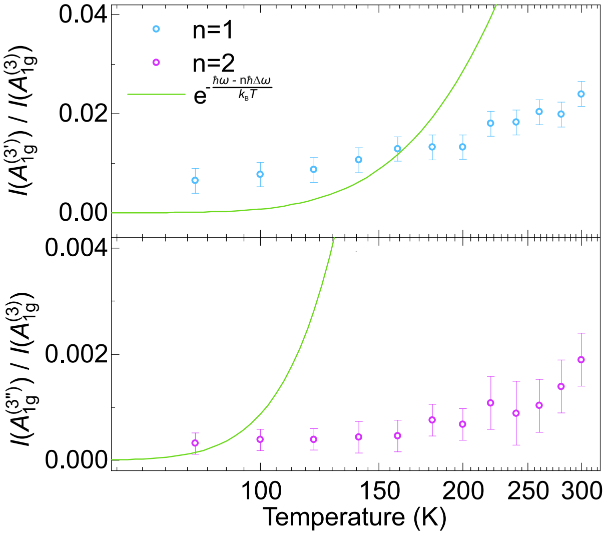

Figure S1. Temperature dependence of the relative integrated intensities of the first ($n = 1$, top panel) and) and second ($n = 2$, bottom panel) satellite peaks of the $A_{1g}^{(3)}$ mode. Symbols denote experimentally extracted intensity ratios $I(A_{1g}^{(3')})/I(A_{1g}^{(3)})$ and $I(A_{1g}^{(3'')})/I(A_{1g}^{(3)})$. Solid lines represent the expected scaling of intensities $I_n/I_0 \propto \exp(-(\hbar\omega - n\hbar\Delta\omega)/k_B T)$ where $\omega = 498.0\ \mathrm{cm}^{-1}$ (at 80 K) and $\Delta\omega = 4.2\ \mathrm{cm}^{-1}$.
